# Knockout of *SlMS10* Gene (*Solyc02g079810)* Encoding bHLH Transcription Factor Using CRISPR/Cas9 System Confers Male Sterility Phenotype in Tomato

**DOI:** 10.3390/plants9091189

**Published:** 2020-09-11

**Authors:** Yu Jin Jung, Dong Hyun Kim, Hyo Ju Lee, Ki Hong Nam, Sangsu Bae, Ill Sup Nou, Yong-Gu Cho, Myong Kwon Kim, Kwon Kyoo Kang

**Affiliations:** 1 Division of Horticultural Biotechnology, Hankyong National University, Anseong 17579, Korea; yuyu1216@hknu.ac.kr (Y.J.J.); skullmask@naver.com (D.H.K.); ju950114@naver.com (H.J.L.); shalom9341@gmail.com (K.H.N.); 2 Institute of Genetic Engineering, Hankyong National University, Anseong 17579, Korea; 3 Department of Chemistry, Hanyang University, Seoul 04763, Korea; sangsubae@hanyang.ac.kr; 4 Department of Horticulture, Sunchon National University, Suncheon 57922, Korea; nis@sunchon.ac.kr; 5 Department of Crop Science, Chungbuk National University, Cheongju 28644, Korea; ygcho@chungbuk.ac.kr; 6 Tomato Research Center, Cheongju 28112, Korea; mkkim207@naver.com

**Keywords:** CRISPR/Cas9, bHLH transcription factor, male sterility, *SlMS10* gene, tomatoes

## Abstract

The utilization of male sterility into hybrid seed production reduces its cost and ensures high purity of tomato varieties because it does not produce pollen and has exserted stigmas. Here, we report on the generation of gene edited lines into male sterility phenotype by knockout of *SlMS10* gene (*Solyc02g079810)* encoding the bHLH transcription factor that regulates meiosis and cell death of the tapetum during microsporogenesis in the tomato. Twenty-eight gene edited lines out of 60 transgenic plants were selected. Of these, eleven different mutation types at the target site of the *SlMS10* gene were selected through deep sequencing analysis. These mutations were confirmed to be transmitted to subsequent generations. The null lines without the transferred DNA (T-DNA) were obtained by segregation in the T_1_ and T_2_ generations. In addition, we showed that the cr-ms10-1-4 mutant line exhibited dysfunctional meiosis and abnormal tapetum during flower development, resulting in no pollen production. RT-PCR analysis showed that the most genes associated with pollen and tapetum development in tomatoes had lower expression in the cr-ms10-1-4 mutant line compared to wild type. We demonstrate that modification of the *SlMS10* gene via CRISPR/Cas9-mediated genome editing results in male sterility of tomato plants. Our results suggest an alternative approach to generating male sterility in crops.

## 1. Introduction

The tomato (*Solanum lycopersicum* L.) is a representative vegetable crop belonging to the *Solanaceae* family, and has high economic value in the market due to its high production and consumption worldwide. In particular, tomatoes can be transformed through *Agrobacterium*-mediated and have been used as a breeding model for horticultural crops due to their relatively short life cycle and small genome size [1]. Tomato seeds are mostly commercially used F_1_ varieties that show a greater biomass, higher disease resistance and greater yield than open pollinated varieties [2]. Plant male sterility is functionally unable to produce or release pollen grains because no anthers, microspores or male gametes are produced [3]. Male tomato sterility has been an interest to many researchers since Crane [4] was first described, and so far approximately 50 male sterility mutants have been reported [5]. It has also been reported that male sterility mutants interfere with division of the tapetum cell, thereby promoting aborted microgametogenesis through genes such as *eme1/exs*, *tpd1*, *ams* and *ms1* [6,7,8]. In *Arabidopsis*, several gene encoding transcription factors have been studied as regulators involved in pollen development such as *AtDYT1, AtTDF1, AtAMS, AtbHLH10, AtbHLH89, AtbHLH91* and *AtMYB103* [9,10,11,12,13,14]. Among transcription factors, the basic helix-loop-helix (bHLH) proteins play an important role in plant growth and development. A total of 152 bHLH transcription factors have been reported in the tomato genome [15,16,17,18,19,20]. The bHLH motif consists of two functionally distinct regions: the basic region for DNA binding and the HLH region for protein dimerization [21].

Depending on DNA-binding ability, proteins that can bind DNA are called DNA-binding bHLH, while others are non-DNA-binding bHLH [22]. Recent studies have shown that non-DNA binding bHLH is functionally very important because heterodimerization occurs through the bHLH domain [23]. It has been reported that the *SlMS10* gene encoding a basic helix-loop-helix transcription factor (bHLH), which carries both programmed cell death and meiosis in the tapetum during microsporogenesis [24]. Molecular marker development and gene targeting have been reported for other genes controlling male sterility such as *ms10*, *ms15*, *ms32*, *ps2* (positional sterile 2), *ex* (exserted stigma) and *7B−1* for tomato breeding [18,19,24,25,26,27]. In that work, male-sterile tomato lines were generated by CRSIPR-CAS9 induced knockout of a stamen-specific gene *SlSTR1* [28]. In a previous study, we succeeded in introducing the male sterility ms10^35^ gene into the elite line (MR10-3211) for backcross breeding [29]. For MAS, the anthocyanin absent (aa) marker for foreground selection and SNP marker derived from resequencing data were used for background selection [16,29]. Therefore, the tomato marker-assisted backcross (MABC) breeding program resulted in a significant reduction of breeding time and cost through a fast selection system compared to conventional breeding. However, these MAS methods have reported that an undesirable genomic segment are linked to a target gene (so-called linkage drag), which causes many breeding difficulties [30]. Recent genome editing techniques have shown the possibility of inducing gene mutations at desired genomic DNA locations using various types of site-specific nucleases [31]. Among them, the CRISPR/Cas9 system has been known to be a powerful genome editing tool in regards to plants and many other organisms [32,33]. The advantage of these systems is the precise and efficient introduction of mutations at the target site. And in the gene edited crops there appears to be no difference in comparison to those developed through conventional breeding techniques. In this study, we focused on the *SlMS10* gene, which encodes the basic helix-hoop-helix transcription factor due to its involvement of programmed cell death and meiosis in the tapetum during microsporogenesis [24]. Therefore, the *SlMS10* gene is suitable as a target gene for generating new male sterility tomato lines by the CRISPR/Cas9 system. Here, we suggest that it overcomes problems inherent in the existing male sterility system and can be easily transferred to new varieties and other crops.

## 2. Materials and Methods

### 2.1. Phylogenetic Tree Analysis

Amino acid sequences of *SlMS10* and other gene homologues related to male sterility were collected from the BLASTP searches (https://blast.ncbi.nlm.nih.gov). Amino acid alignments were obtained by ClustalW of MEGA 7.0 through default parameters. A total of 37 full-length amino acid sequences from plant species were aligned and the maximum likelihood tree was created with default settings. A phylogenetic tree was constructed by neighbor-joining method according to what was previously reported by Kumar [34]. Table S1 shows the sequence IDs of all gene sequences.

### 2.2. sgRNA-Cas9 Vector Construction

For the design of sgRNAs, three target sites were first selected using the CRISPR RGEN tool program (http://www.rgenome.net/) from the *SlMS10* sequence (Table S2). The T7E1 assay was performed in the same way as previously reported by Jung [33]. In the pBAtC vector, the Cas9 gene was regulated by the 35S mosaic virus promoter, and the sgRNA was controlled by the *Arabidopsis* U6 promoter [33]. DNA oligos corresponding to the designed sgRNAs were synthesized by Bioneer co., Ltd. (Dajeon, Korea) (Table S7) and the dimer was cloned into plant expression vector, pBAtC. The constructed plasmid, *AtU6*:sgRNA/pBAtC was introduced into the *A. tumefaciens* strain EHA105 using electroporation method [35].

### 2.3. Transformation into Tomato

The transformation procedure was carried out with KS-13 variety (wild type) as described previously by Jung [36]. Briefly, cotyledons of 10~14-day old seedlings were immersed in *Agrobacterium* suspension culture, gently stirred and blotted on sterilized Whatman paper. The slightly dried explants were transferred to a co-culture medium containing; 4.3 g/L MS medium, 30.0 g/L sucrose, 300 mg/L zeatin, 30 mg/L acetosyringone and 3.0 g/L gelrite, and incubated for 2 days in darkness at 23 ± 2 °C. After co-culture, all explants were washed with sterile distilled water containing 300 mg/L cefotaxime to prevent overgrowth of bacteria. These washing procedures were repeated 3 times and then blotted on sterile Whatman paper. The leaf discs were transferred on selection medium enriched with 4.3 g/L MS medium, 30.0 g/L sucrose, 300 mg/L zeatin, 400 mg/L carbenicillin, 100 mg/L kanamycin and 3.0 g/L gelrite and placed it upside down. Regenerated shoots (2 cm in height) were separated from the original explants and transferred to rooting medium containing 0.3 mg/L IAA, 50 mg/L kanamycin and 400 mg/L carbenicillin. Tomato plants with well-developed roots were acclimatized and transferred to pots and grown until maturity in a greenhouse.

### 2.4. Targeted Deep Sequencing and Mutation Analysis

DNA extraction from tomato plants was performed using the DNA Quick Plant Kit (Inclone Ltd., Seoul, Korea). First, to identify transformants from regenerated plants, PCR analysis was performed using NPTII gene-specific primers. Then, targeted deep sequence analysis was performed as described by Jung [33]. A list of all primers used for targeted deep sequencing was shown in Table S2. Paired-end read sequencing by PCR amplicons was produced with MiniSeq (Illumina, San Diego, CA, USA). All data derived from sequences was analyzed by Cas-Analyzer (http://www.rgenome.net/cas-analyzer), as previously reported by [37]. It is commonly known that in the CRISPR/Cas9 system, the Cas cleavage site occurs mostly 3 bp upstream of the protospacer. Therefore, the insertion and deletion mutations around 3 bp upstream of the protospacer were considered to be mutations induced by Cas9. Transgene-free mutant plants were screened in T_1_ generation and double confirmed in the T_2_ generation. To obtain transgene-free plants, PCR amplification was performed using DNA extracted from individual plants using NPTII gene-specific primers. For the potential off-target analysis, PCR analysis was performed by specific primers using transgene free plants (Tables S6 and S7). PCR products were sequenced and confirmed for mutation.

### 2.5. Plant Growth and Morphological Characterization

All tomato seedlings were cultivated in pots using sterilized soil in the greenhouse of a farm attached to Hankyong National University (Ansung, Korea). Flower traits were investigated in the wild type (WT) and mutant lines of T_1_ and T_2_ generation. Two flowers per plant were collected using a stereoscopic microscope, and flower morphology was observed at the anthesis stage. The number of sepals and petals was counted for each flower, and the length and width were measured using representative sepals and petals. Also, anther, ovary and pistil length were measured. All the recorded data were showed as mean ± standard error of the mean (SEM).

### 2.6. Microscopy

To determine the pollen phenotype, two flowers per plant were sampled, stained with 1% acetic acid acetic acid solution and evaluated by light microscopy. For the paraffin section, floral buds around the meiotic stage were harvested from each plant. First, floral buds were treated with 15% hydrofluoric acid, followed by dehydration, removal, infiltration and embedding. For imaging, a 10 μm microtome section was placed on glass slides and floated in a 37 °C water bath containing deionized water. The sections were floated onto clean glass slides and microwaved at 65 °C for 15 min. Then, the tissue was bound to the glass. Each slide was used in chemical staining immediately. To distinguish the floral phenotype, scanning electron microscopy (SEM) was investigated as reported by Mazzucato [38]. The explants were prepared by removing sepals and petals, and after separating the individual anthers from androecium, they were fixed in 3% glutaraldehyde (pH 7.2) for 3 h, rinsed in cacodylate buffer for 10 h, post-fixed in 1.1% osmium tetroxide for 8 h and dehydrated with a graded ethanol series. The floral phenotype was observed by a 5200 JEOL JSMSEM (JEOL Ltd., Tokyo, Japan).

### 2.7. RNA Isolation and RT-PCR Analysis

Total RNA was isolated from plant leaves using Trizol reagent (Invitrogen, Seoul, Korea). cDNA synthesis was performed by reverse transcriptase (Promega, Seoul, Korea) using 2 μg of total RNA. For RT-PCR analysis, 200 ng of cDNA was used, and the amplified PCR product was separated on a 1% agarose gel. Then, it was stained with ethidium bromide and photographed under a UV lamp. The primer sequences of genes used for the RT-PCR analysis are shown in Table S7.

## 3. Results

### 3.1. Phylogenetic Analysis of SlMS10 and Other bHLH Homologues

**Figure 1:**
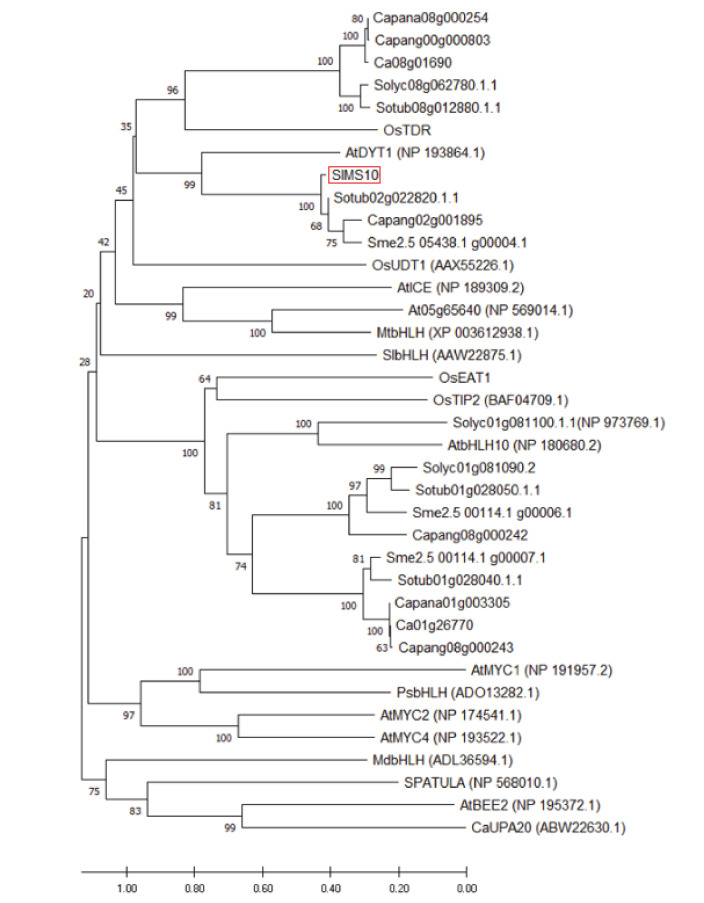
Phylogenetic analysis of bHLH proteins in several plant species. The sequence IDs of all gene sequences are shown in Supplementary Table S1. The phylogenetic tree was constructed by the neighbor-joining method, using MEGA. The numbers represent bootstrap values from 1000 replicates (https://www.megasoftware.net/home).

Phylogenetic analysis was performed to obtain information about the genetic relationship between *SlMS10* and other bHLH homologues related to the male sterility of other plant families. *SlMS10* and other bHLH homologues with thirty-seven genes collected from the NCBI database was investigated and analyzed using the MEGA7 program (Figure 1). As shown in Figure 1, phylogenetic numbers indicated that all estimated and identified proteins of *SlMS10* and other *bHLH* homologues were classified as clades. From the phylogenetic tree, three proteins related to *SlMS10* and bHLH homologues were recorded in the tomato genome and are well conserved in plant genomes of different species (Figure S1). The results showed that *SlMS10*, *OsUDT1*, *CabHLH* and *AtDYT1* were classified within the same clade (Figure 1 and Figure S1). These results suggested that the transcription factors of tomato bHLH members showed very high homology regardless of origin.

### 3.2. Generation of Male Sterility Lines by CRISPR/Cas9 System

**Figure 2:**
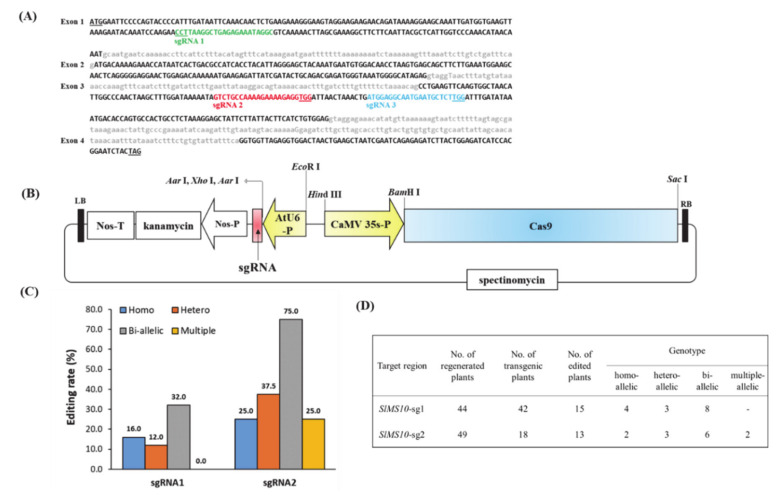
Genome editing of the *SIMS10* gene encoding bHLH transcription factor using CRISPR/Cas9 system. (**A**) Target sequence in the *SIMS10* gene of each sgRNA. Green, red, blue color are sgRNAs. Underlined is the PAM site. (**B**) The structure of the T-DNA region of a Cas9/single guide RNA (sgRNA) vector. Marker gene phosphinothricin (PPT) was driven by the Nos promoter, whereas the sgRNA was driven by the *Arabidopsis* U6 promoter and the Cas9: NLS was driven by the CaMV-35S promoter. LB left border, RB Right border. (**C**) Efficient ratio for homo-, hetero-, bi- and multi-allelic in genome editing in T_0_ generation. (**D**) Frequency of gene editing for sgRNA 1 and sgRNA2 respectively.

**Figure 3:**
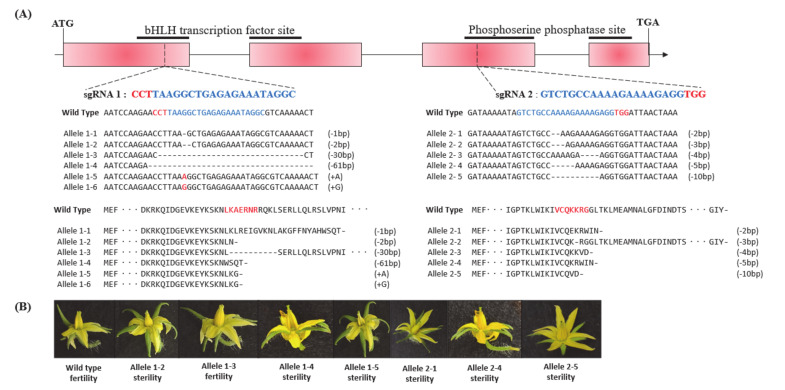
Mutation pattern and flower phenotype according to sgRNA locus. (**A**) Schematic representation of CRISPR/Cas9-mediated targeted mutagenesis in *SlMS10* gene. The sgRNA and PAM site are represented in blue and red. The wild-type and mutations generated of the gene coding sequences are shown. (**B**) Flower phenotypes of edited plants and WT (wild-type).

To generate transgenic plants with targeted mutations in the *SlMS10* gene, CRISPR/Cas9 vector constructs targeting the first and third exons of the *SlMS10* gene were designed, respectively (Figure 2A and Table S2) and used for transformation. Two target sites, sgRNA1 and sgRNA2, were designed for *SlMS10* (Figure 2A), and 60 T_0_-independent transgenic tomato plants were generated from hypocotyls of tomato inbred KS-13 line using *Agrobacterium*-mediated transformation. Furthermore, chimeric, biallelic, heterozygous and homozygous *SlMS10* mutants were present in the T_0_ generation (Figure 2B–D and Table S3). Most of the mutants displayed a frame-shift type exception of some mutants where in-frame deletion occurred in the target site (Figure 3A). In addition, male sterility in shift frame mutants and in-frame mutants was investigated. As a result, the in-frame mutants normally produced pollens, but all mutants with shift frames showed male sterility (Figure 3B and Figure S2). Among the shift frame mutants, the “Allele 1-4” and “Allele 2-5” deletion mutants in the target site were selected and referred to as cr-ms10-1-4 (-61/-61) and cr-ms10-2-8 (-10/-10), respectively. The selected mutants were crossed with the KS-13 pollen used for transformation to generate T_1_ seeds. The transgene-free T_1_ and T_2_ mutant lines were screened by Mendel’s law and potential off-target mutations in the T_2_ mutant line were investigated (Figure S2D). Ten potential off-target sites including four mismatched bases were examined using the Cas-OFFinder (http://www.rgenome.net/cas-offinder/) [39] (Table S6). PCR products obtained from T_2_ mutant plants without transgene were sequenced. No mutations were seen in all 10 potential off-target sites, indicating mutagenesis of the predicted site is designed with high specificity (Table S4).

### 3.3. Phenotypic Characterization of the cr-ms10-1-4 and cr-ms10-2-8 Mutant Lines

**Figure 4:**
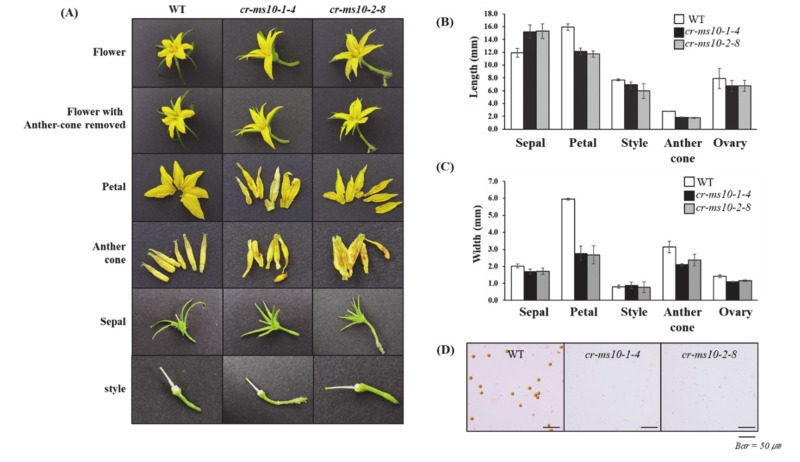
Morphometric data of flowers from edited lines (cr-ms10-1-4 and cr-ms10-2-8) and WT plants (**A**) Morphology of each flower organs in WT, cr-ms10-1-4 and cr-ms10-2-8 line. (**B**,**C**) Length and Width of floral organs in WT, cr-ms10-1-4 and cr-ms10-2-8 line. (**D**) Analysis of pollen viability by acetocarmine staining.

Phenotypes of the cr-ms10-1-4, cr-ms10-2-8 mutant lines and WT plants were almost similar until the flowering stage (Figure 4A). However, the cr-ms10-1-4 and cr-ms10-2-8 mutant lines had longer sepals and shorter petals than the WT plants at the flowering stage (Figure 4A). In addition, the stamens of the cr-ms10-1-4 and cr-ms10-2-8 flowers are significantly reduced, have a bright color and, in general, the stigma remains strong (Figure 4B,C and Table S5). As a result of staining with 1% acetocarmine to confirm the pollen viability analysis, pollen was not detected in the cr-ms10-1-4 and cr-ms10-2-8 lines, although it appeared normal in WT plants (Figure 4D). Therefore, the cr-ms10-1-4 and cr-ms10-2-8 lines could not produce fruit sets after their self-pollination, but were able to produce fruit by manually pollinating the pollen of the WT plant. The results of the cr-ms10-1-4 and cr-ms10-2-8 lines generated using the CRISPR/Cas9 system were previously consistent with the results of several researchers [32,36].

### 3.4. Histological Examination of Anthers to cr-ms10-1-4 Line

**Figure 5:**
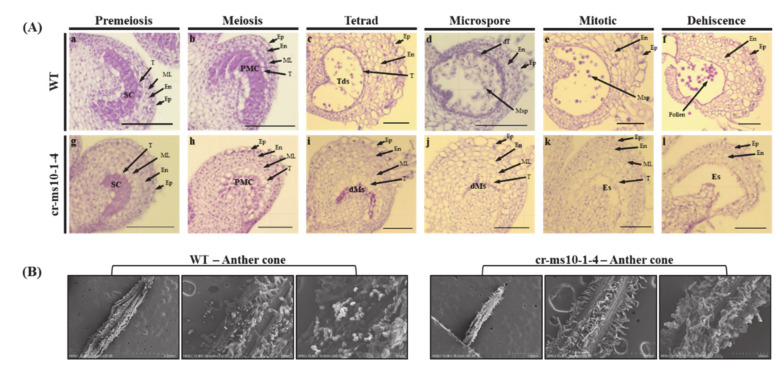
Histological examination of anthers at different developmental stages. (**A**) Transverse sections of WT (**a**–**f**) and cr-ms10-1-4 (**g**–**l**) anthers at different developmental stages. (**a**,**g**) Premeiotic stage; (**b**,**h**) Meiotic stage; (**c**,**i**) Tetrad stage; (**d**,**j**) Microspore stage; (**e**,**k**) Mitotic stage; (**f**,**l**) Dehiscence stage. dMs, degenerated meiocytes; dT, degenerated tapetum; En, endothecium; Ep, epidermis; ML, middle cell layer; Msp, microspore; PMC, pollen mother cell; SC, sporogenous cell; T, tapetum; Tds, tetrads. Scale bars, 50 μm. (**B**) SEM analysis between cr-ms10-1-4 line and WT.

To investigate the spatial and temporal occurrences of defects in the cr-ms10-1-4 lines, we performed paraffin sections and a histological examination on anthers at different developmental stages (Figure 5A). In the preliminary meiosis stage, cell layer differentiation in the anthers of the cr-ms10-1-4 lines appeared to be similar to that in WT anthers. In the meiosis phase, meiosis was completed when spore cells developed from pollen mother cells (PMCs) (Figure 5A(b,h)). From this point on, morphological differences were observed between cr-ms10-1-4 and WT anther. In WT anthers, PMCs were divided into successive tetrads after meiosis and continuously developed into microspores, vacuolated microspores and pollen grains (Figure 5A). In addition, tapetal cells were highly condensed, deeply stained and gradually disappeared. However, PMCs in cr-ms10-1-4 anthers were crushed and could not produce tetrads (Figure 5A(c,i)). In addition, the tapered cells were over-expanded and vacuolated at the tetrad phase and maintained until the dehiscence phase (Figure 5). Additionally, cr-ms10-1-4 and WT anthers were observed by SEM analysis. As a result, WT anthers appeared to be normal globular pollen grains, but no cr-ms10-1-4 anther was observed (Figure 5B).

### 3.5. Expression Analysis of Genes Related Floral Development

**Figure 6:**
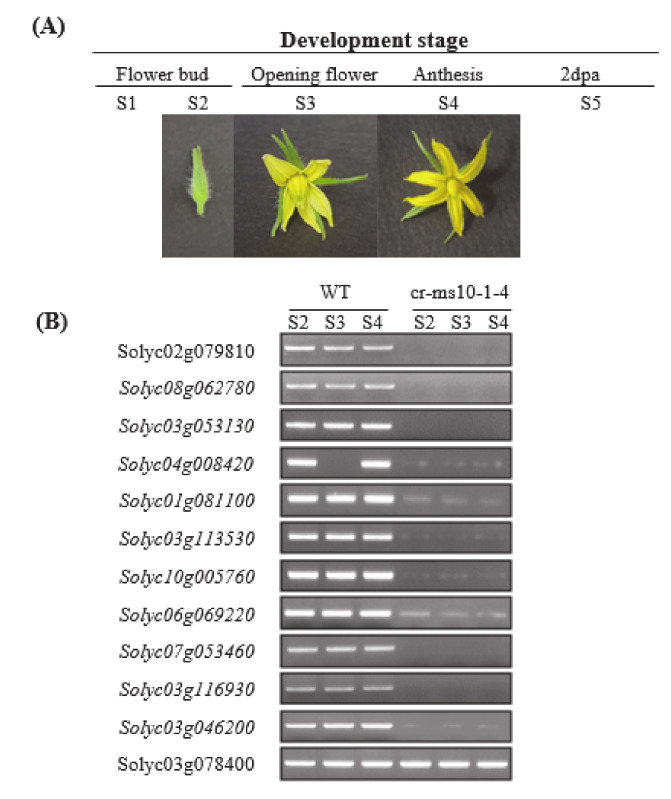
(**A**) Developmental stages of tomato flowers in wild-type. dpa: days post anthesis. (**B**) Relative expression levels of cr-ms10-1-4 compared to that of WT and actin detected by quantitative RT-PCR. *Solyc02g079810, MS10*; *Solyc08g062780*, *AMS-like*; *Solyc03g053130, SlSTR1*; *Solyc04g008420*, *AMS-like-1*; *Solyc01g081100, MS32*; *Solyc03g113530*, *AtTDF1-like*; *Solyc10g005760*, *MYB103-like*; *Solyc06g069220*, *Aspartic protease-1*; *Solyc07g053460*, *Cysteine protease*; *Solyc03g116930*, Sister chromatid cohesion; *Solyc03g046200*, *Endo-1,3-beta-glucanase*; *Solyc03g078400*, *Actin*. Oligonucleotide primers are described in Supplementary Table S7.

Considering that the cr-ms10-1-4 mutant line cannot produce pollen due to abnormality in meiosis and tapetum development (Figure 6A), analysis of the expression of ten gene-related flower developments using RT-PCR analysis was performed (Figure 6B). First, genes such as *Solyc03g116930* encoding sister chromatid cohesion, *Solyc07g053460* encoding cysteine protease, *Solyc08g062780* encoding AMS-like and *Solyc03g053130* encoding *SlSTR1* were strongly expressed in WT, but did not express in the cr-ms10-1-4 mutant line (Figure 6B). Also, the expression levels of genes such as *Solyc01g081100*, *Solyc03g113530*, *Solyc03g059200*, *Solyc06g069220*, *Solyc03g046200*, *Solyc02g079810* and *Solyc04g008420* in the cr-ms10-1-4 mutant line were very low compared to WT (Figure 6B and Figure S3).

## 4. Discussion

**Figure 7:**
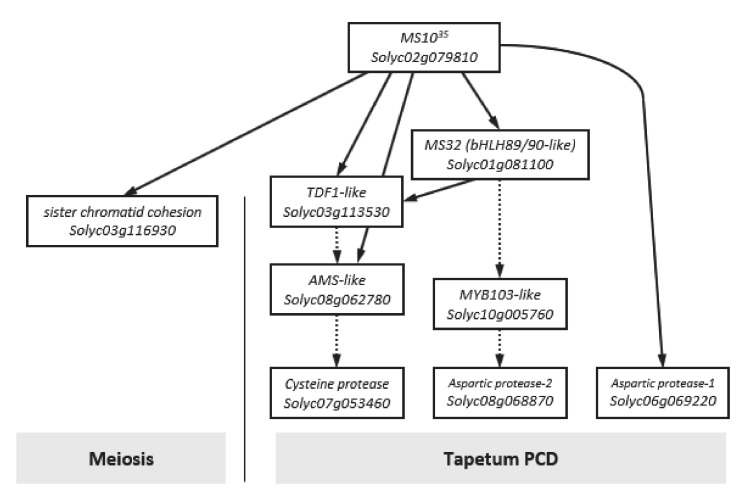
A model for anther development regulated in tomato and *Arabidopsis*. Solid arrows represent regulation in tomato, and dotted arrows indicate regulation in *Arabidopsis*. PMC, pollen mother cell; PPC, primary parietal cell; PSC, primary sporogenous cell; SPC, secondary parietal cell.

So far, one of the most widely used tools in plant breeding is production of the F_1_ hybrid seed by heterosis mechanism, which has advantages in terms of increased productivity, environmental suitability and disease resistance. For many horticultural crops such as tomatoes, carrots and peppers, the costs of seed production and labor increase due to the emasculation process of manually removing anthers from female flowers [40]. In addition, the application of male sterility is a very efficient approach to reducing hybrid seed costs and ensuring high varietal purity [2]. Since the first description by Crane [4], tomato male sterility has been an interest for many researchers, and so far about 50 male sterility mutants have been reported [5,15,16,17,18,19,24,41,42,43,44]. These spontaneous male-sterile mutants are an excellent system for integrating male sterility for hybrid seed production. However, to date, male sterility in tomato breeding has not been utilized in hybrid seed production because it is a natural mutant. Recently, genome editing techniques have shown the capability of reducing heritable mutations at desired genome position by using various types of site-specific nucleases [31]. Of these, the CRISPR/Cas9 system quickly emerged as a powerful genome editing tool in many organisms, including crops [32,45]. In the genome editing system, mutation is introduced accurately and efficiently at the target site, and the modified crops have the advantage that they are no different from those developed by common breeding techniques. In this study, we established tomato lines in which the coding regions of bHLH transcription factor were deleted from the *SlMS10* gene via a genome editing procedure. Tomatoes contain four bHLH genes, of which the *SlMS10* gene is known for its strong expression in tapetum tissues [19], implying that *SlMS10* is a possible target for editing technology for male sterility. In addition, Figure 1 shows that *SlMS10* has an authentic bHLH, as can be seen in most plant bHLHs, meaning pollen and tapetum development is regulated. For the mutagenesis, we performed editing at the target site of the coding region for *SlMS10* using the CRISPR/Cas9 system. Out of 28 T_0_ transgenic plants, eleven edited plants were generated (Figure 2 and Table S3). Out of 28 plants analyzed at the DNA level, 22 plants contained the expected deletion (82%) (Figure S2). Together with them, we selected 2 transgene-free homozygous knockout lines, including cr-ms10-1-4 and cr-ms10-2-8, which themselves showed coding frame shifts and premature translational stops in the T_1_ and T_2_ generations (Figure 4). The remaining lines were mostly single base deletions and insertions (Figure 3). Jung [33] reported the deletion of a target gene in tomatoes via CRISPR/Cas9, and the frequency of deletion correlated with the target size. For example, it was about 67% for 1 to 10 bp deletion, but only 3% for 10 bp or higher deletion. It is clear that shorter deletions such as 1 bp or 2 bp appear at high frequency. In addition, some lines of T_1_ generation contained two or three mutations, including a wild-type, to represent a bi-allelic, as well as chimeric form. The cr-ms10-1-4 and cr-ms10-2-8 mutant lines obtained in this experiment had longer sepals and shorter petals than the WT plant at the flowering stage. These lines did not release pollen from flowers during anthesis (Figure 4). Paraffin section examination showed defects in microgametogenesis and tapetum degradation due to the *SlMS10* gene knockout in the cr-ms10-1-4 lines. After reaching the tetrad phase, the anthers of the cr-ms10-1-4 lines degenerated PMCs and failed to perform a series of processes such as tetrads, microspores and pollen grains (Figure 5A(c,i)). Thus, the tapetal cells were abnormally expanded and remained empty without degeneration (Figure 5A,B). In addition, RT-PCR analysis showed that most of the genes proposed to be involved in pollen and tapetum development in tomatoes were either not expressed or weakly expressed in the cr-ms10-1-4 lines (Figure 6). In previous studies of *Arabidopsis* and rice, two pathways have been reported that control pollen and tapetum development [14,24,45,46]. So far, two genes are known to regulate the development of pollen and tapetum in tomatoes, of which *SlMS10* has a homolog to *AtDYT1* and *OsUDT1*, while another was *Solyc01g081100*, which has a homolog of *AtbHLH10/89/90* and *OsEAT1* [19,24]. It has been reported that the loss of function of these genes in tomatoes results in low expression of the transcription factor genes such as *AtTDF1-like*, *AtAMS-like*, *AtMYB103-like* and *AtMS1-like* (Figure 7) [47]. In our experiment, meiosis-related genes, tapetum-specific genes and transcription regulatory genes were strongly expressed in WT, but did not express in the cr-ms10-1-4 mutant line (Figure 6B). Also, the expression levels of genes such as *Solyc01g081100*, *Solyc03g113530*, *Solyc03g059200*, *Solyc06g069220*, *Solyc03g046200*, *Solyc02g079810* and *Solyc04g008420* in the cr-ms10-1-4 mutant line were very low compared to WT (Figure 6). In rice, the *OsEAT1* gene has also been reported to activate tapetum cell death by regulating aspartic acid protease. However, the aspartic protease gene did not show significantly different expression in the cr-ms10-1-4 mutant lines (Figure 6). Thus, our gene editing studies selected the cr-ms10-1-4 elite line with male sterility, as well as to aid in understanding the details of regulating pollen and tapetum development in tomatoes. Through this study, male sterility was generated via the CRISPR/Cas9 system, which can be quickly introduced into the elite lines, eliminating linkage drags and shortening the required time compared to conventional breeding methods. The CRISPR/Cas9 system can be applied to other horticultural crops if a region conserved in stamen-specific genes is used as sgRNAs.

## 5. Conclusions

The utilization of male sterility in hybrid seed production reduces its cost and ensures high purity of tomato varieties because it produces no pollen and has exserted stigmas. Twenty-eight gene edited lines out of 60 transgenic plants were selected. Of these, eleven different mutation types at the target site of the *SlMS10* gene were selected via deep sequencing analysis. These mutations were confirmed to be transmitted to subsequent generations. The null lines without the transferred DNA (T-DNA) were obtained by segregation in the T_1_ and T_2_ generations. In addition, we showed that the cr-ms10-1-4 mutant line exhibited dysfunctional meiosis and abnormal tapetum during flower development, resulting in no pollen production. RT-PCR analysis showed that the most genes associated with pollen and tapetum development in tomatoes had lower expression in the cr-ms10-1-4 mutant line compared to wild type. We demonstrate that modification of the *SlMS10* gene via CRISPR/Cas9-mediated genome editing results in male sterility of tomato plants. Our results suggest an alternative approach to generating male sterility in crops.

## Supplementary Materials

The following are available online at https://www.mdpi.com/2223-7747/9/9/1189/s1. Figure S1. Multiple sequence alignments of *SlMS10* proteins identified. XP_025885203.1, *Solanum lycopersicum* XP_006365867.2, *Solanum tuberosum*; XP_015065394.1, *Solanum pennellii*; XP_016449679.1, *Nicotiana tabacum*; XP_016562081.1, *Capsicum annuum*. Consensus keys: ‘*’, single, fully conserved residue; ‘:’, conservation of strong groups; ‘.’, conservation of weak groups; ‘-’, no consensus. Figure S2. (A) Gel electrophoresis of PCR products amplified from putative transgenic plants. (B) Phenotypes of edited plants (C) Fruit shape of T_1_ generation obtained by crossing between edited plants and KS-13 lines (D) Selection of null segregant in T_1_ and T_2_ generation by PCR analysis. No band indicated T-DNA free plants. Figure S3. Relative expression levels of cr-ms10-1-4 compared to that of WT and actin detected by quantitative RT-PCR. *Solyc02g079810*, *MS10*; *Solyc08g062780*, *AMS-like*; *Solyc03g053130*, *SlSTR1*; *Solyc04g008420*, *AMS-like-1*; *Solyc01g081100*, *MS32*; *Solyc03g113530*, *AtTDF1-like*; *Solyc10g005760*, *MYB103-like*; *Solyc06g069220*, *Aspartic protease-1*; *Solyc07g053460*, *Cysteine protease*; *Solyc03g116930*, Sister chromatid cohesion; *Solyc03g046200*, *Endo-,3-beta-glucanase*; *Solyc03g078400*, *Actin*. Table S1 Amino acid sequences of *SlMS10* and other gene homologues related to male sterility investigated in this study. Table S2 Design of sgRNAs for CRISPR genome editing on *SlMS10* gene in tomato using the CRISPR RGEN tool program (http://www.rgenome.net/). Table S3 Frequency of genome editing of *SlMS10* gene using CRISPR/Cas9 system. Table S4 Detection of mutations on the putative off-target sites in edited plants. Table S5 Variation of length and width of each flower organs in edited lines (cr-ms10-1-4 and cr-ms10-2-8) and wild type. Table S6 Oligonucleotide primers of the putative off-target sites for mutation analysis in edited plants. Table S7 Oligonucleotide primers used for recombinant vector construction, deep sequencing and RT-PCR analysis in these studies.
