# Supplementary material for: Knockout of SlMS10 Gene (Solyc02g079810) Encoding bHLH Transcription Factor Using CRISPR/Cas9 System Confers Male Sterility Phenotype in Tomato
Source: Plants (Basel). 2020 Sep 11;9(9):1189. doi: 10.3390/plants9091189 (PMC7570381; doi:10.3390/plants9091189)
Supplement: Supplementary file 1 [file plants-09-01189-s001.pdf]

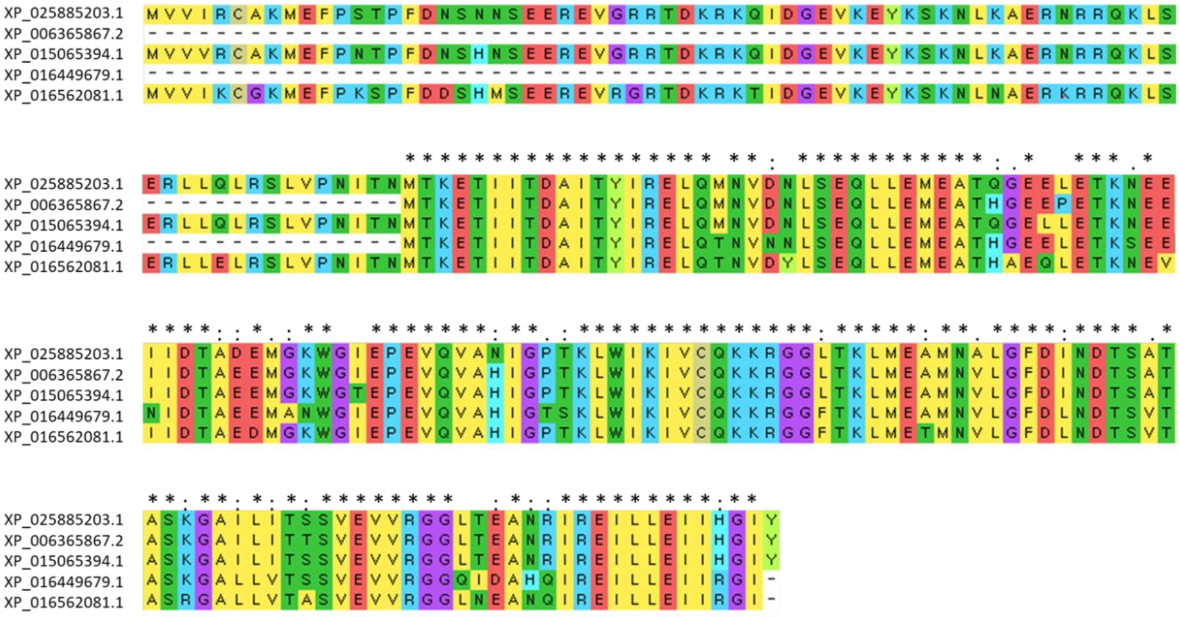

Figure S1. Multiple sequence alignments of *SlMS10* proteins identified. XP_025885203.1, *Solanum lycopersicum* XP_006365867.2, *Solanum tuberosum*; XP_015065394.1, *Solanum pennellii*; XP_016449679.1, *Nicotiana tabacum*; XP_016562081.1, *Capsicum annuum*. Consensus keys: '*', single, fully conserved residue; ':', conservation of strong groups; '.', conservation of weak groups; '-', no consensus.

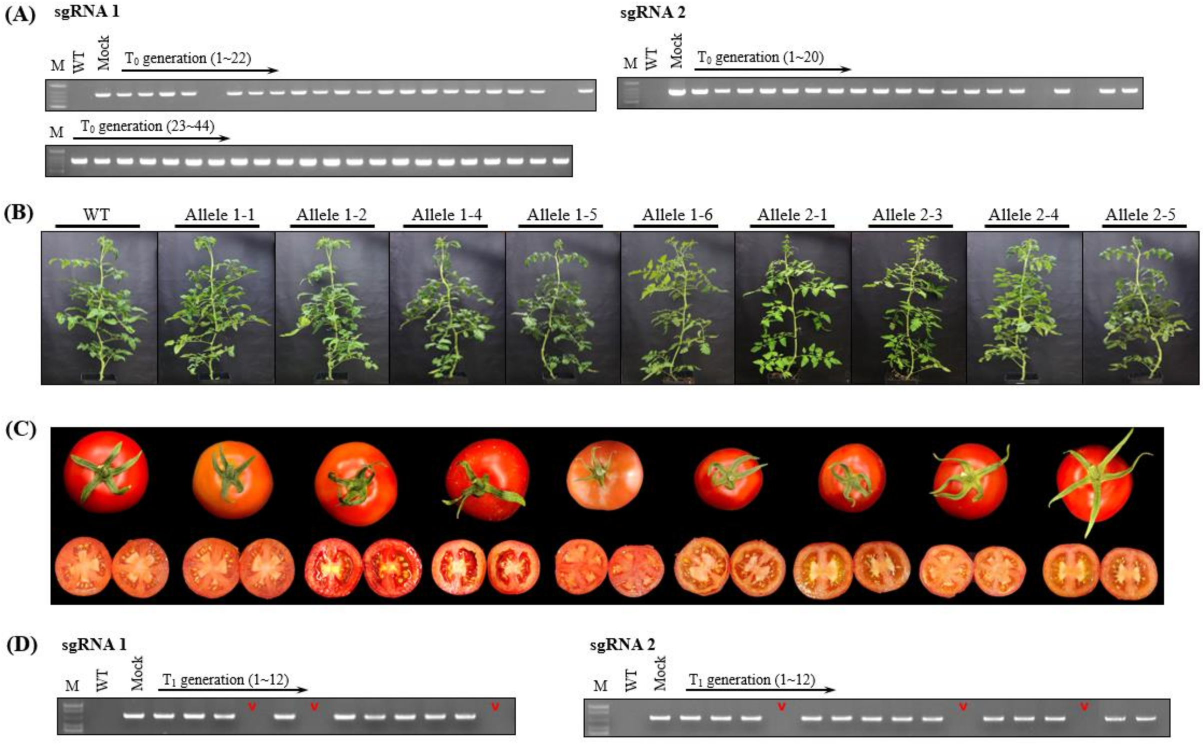

Figure S2. (**A**) Gel electrophoresis of PCR products amplified from putative transgenic plants. (**B**)Phenotypes of edited plants (**C**) Fruit shape of T_1 generation obtained by crossing between edited plants and KS-13 lines (**D**) Selection of null segregant in T_1 and T_2 generation by PCR analysis. No band indicated T-DNA free plants.

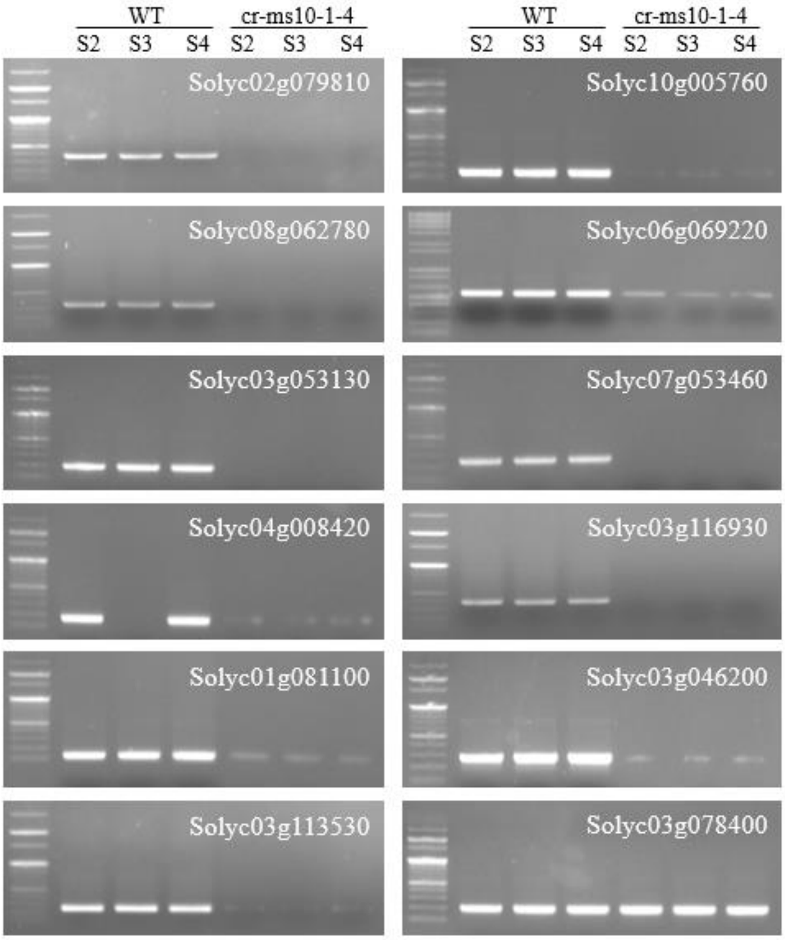

Figure S3. Relative expression levels of cr-ms10-1-4 compared to that of WT and actin detected by quantitative RT-PCR. Solyc02g079810, MS10; Solyc08g062780, AMS-like; Solyc03g053130, SlSTR1; Solyc04g008420, AMS-like-1; Solyc01g081100, MS32; Solyc03g113530, AtTDF1-like; Solyc10g005760, MYB103-like; Solyc06g069220, Aspartic protease-1; Solyc07g053460, Cysteine protease; Solyc03g116930, Sister chromatid cohesion; Solyc03g046200, Endo-,3-beta-glucanase; Solyc03g078400, Actin.

Table S1. Amino acid sequences of *SlMS10* and other gene homologues related to male sterility investigated in this study.

Species	Identifier	GenBank number
Arabidopsis thaliana	bHLH DNA-binding superfamily protein	NP_568010.1
	bHLH DNA-binding superfamily protein	NP_172107.1
	bLH protein 93	NP_569014.1
	BR enhanced expression 2	NP_195372.1
	bHLH DNA-binding superfamily protein	NP_180680.2
	bHLH DNA-binding superfamily protein	NP_193864.1
	bHLH DNA-binding superfamily protein	NP_001030774.1
	bHLH DNA-binding superfamily protein	NP_191957.2
	bHLH DNA-binding family protein	NP_174541.1
	bHLH DNA-binding family protein	NP_193522.1
Capsicum annuum	BTB/POZ domain-containing protein-like	KAF3625311.1
	transcription factor ABORTED MICROSPORES-like isoform	KAF3650000.1
	BTB/POZ domain-containing protein-like	KAF3625311.1
	transcription factor ABORTED MICROSPORES isoform	XP_016537577.1
	transcription factor ABORTED MICROSPORES isoform	XP_016537580.1
	flavoprotein wrbA-like	KAF3666794.1
	3-hydroxyisobutyryl-CoA hydrolase 1-like	KAF3625310.1
	bHLH transcription factor Upa20	ABW22630.1
Capsicum chinense	protein BC332_02983	PHU30890.1

Malus domestica	transcription factor bHLH106-like	NP_001281284.1
Medicago truncatula	transcription factor bHLH93 isoform	XP_003612938.1
Oryza sativa Japonica Group	transcription factor EAT1	XP_015634639.1
	transcription factor TDR	XP_015625730.1
	transcription factor TIP2	XP_015645943.1
	undeveloped tapetum 1	AAX55226.1
Pisum sativum	Basic helix-loop-helix protein A	E3SXU4.1
Solanum lycopersicum	bHLH91-like	XP_004229579.1
	transcription factor ABORTED MICROSPORES isoform X2	XP_004245356.1
	transcription factor DYT1	XP_025885203.1
	bHLH transcriptional regulator	NP_001234654.2
Solanum pennellii	transcription factor DYT1	XP_015065394.1
	transcription factor bHLH91-like	XP_015087359.1
Solanum tuberosum	transcription factor bHLH91-like	XP_006354586.1
	transcription factor DYT1	XP_006365867.2
	transcription factor ABORTED MICROSPORES isoform	XP_006351593.1
	transcription factor bHLH91-like	XP_006354586.1
	transcription factor bHLH89-like	XP_006354670.1

Table S2. Design of sgRNAs for CRISPR genome editing on *SlMS10* gene in tomato using the CRISPR RGEN tool program (http://www.rgenome.net/).

Ms10	RGEN Target (5' to 3')	Direction	GC content (%, w/o PAM)	Out-of frame score	Mismatches			
					0	1	2	3
sgRNA1	GCCTATTTCTCTCAGCCTTAAGG	−	45.0	68.9	1	0	0	0
sgRNA2	GTCTGCCAAAAGAAAAGAGGTGG	+	45.0	73.1	1	0	0	0
sgRNA3	ATGGAGGCAATGAATGCTCTTGG	+	45.0	67.0	1	0	0	0

Table S3. Frequency of genome editing of *SlMS10* gene using CRISPR/Cas9 system.

Target region	No. of regenerated plants	No. of transgenic plants	No. of edited plants	Genotype			
				homo-allelic	hetero-allelic	bi-allelic	Multiple-allelic
SlMS10-sg 1	44	42	15	4	3	8	−
SlMS10-sg 2	20	18	13	2	3	6	2

Table S4. Detection of mutations on the putative off-target sites in edited plants.

Target	Putative off-target	Off-target locus	Sequence of off-target site	No. of mutation plants
sgRNA 1	OFF 1	ch02:611807-611829	GCCTATTgaTtTCAaCCTTAGGG	0
	OFF 2	ch03:2207687-22076709	GCCTAgTTCTtgCAGCtTTATGG	0
	OFF 3	ch03:9357140-9357162	GCCTATTgtaCaCAGCCTTACGG	0
	OFF 4	ch09:1577070-157729	GaggATTTCTCTtAGCCTTAGGG	0
	OFF 5	ch09:58165589-58165611	cCaTATTTCTCTCAagCTTAAGG	0
	OFF 6	ch12:6683409-6683409	aCCTATTTCTtTCAGgtTTATGG	0
sgRNA 2	OFF 1	ch01:56429029-56429051	tcCTGCaAAAAGAAAAGAaG	0
	OFF 2	ch01:74607309-74607931	ccCTGCaAAAAGAAAAGAaG	0
	OFF 3	ch03:29122126-29122148	aTCTGagAAAAGAgAAGAGG	0
	OFF 4	ch07:6556684-6556706	GTCTtttAAAAGAAAAGAcG	0
	OFF 5	ch10:42400100-42400122	agCTGCCAAAAGAAAAGgGa	0

Table S5. Variation of length and width of each flower organs in edited lines (cr-ms10-1-4 and cr-ms10-2-8) and wild type.

Flower organs	WT (mm)	cr-ms10-1-4 (mm)	cr-ms10-2-8 (mm)
Sepal length	24.14 ± 1.65a	13.54 ± 1.06b	14.15 ± 1.15b
Sepal width	2.01 ± 0.12a	1.69 ± 0.16b	1.72 ± 0.20b
Petal length	15.95 ± 0.52a	12.15 ± 0.52b	13.47 ± 0.50b
Petal width	6.00 ± 0.06a	2.77 ± 0.41b	2.68 ± 0.53b
Style length	7.69 ± 0.13a	6.92 ± 0.47b	5.96 ± 1.17b

Style width	0.70 ± 0.09a	0.88 ± 0.19a	0.77 ± 0.33a
Ovary length	2.78 ± 0.02a	1.85 ± 0.05b	1.78 ± 0.09b
Ovary width	3.02 ± 0.34a	2.09 ± 0.09b	2.37 ± 0.34b
Anther cone length	7.92 ± 1.59a	6.76 ± 0.88b	6.79 ± 0.84b
Anther cone width	1.31 ±0 .09a	1.08 ± 0.03b	1.15 ± 0.04b

Means ± standard deviation (±sd.), n = 3. Statistical differences among the agronomic traits were detected by Duncan's multiple range test ($p < 0.05$).

Table S6. Oligonucleotide primers of the putative off-target sites for mutation analysis in edited plants.

Off-target site	Primers	Sequence (5'→3')
sgRNA 1 OFF1	FW	TTTAATAATCATCTCGTCTGGTCA
	RV	TGTTGATTATAATTGTTTATC
sgRNA 1 OFF2	FW	TTGTGCAGCTGCTTTCTAATTC
	RV	TAAATAAAACCATTTATTTGAACAT
sgRNA 1 OFF3	FW	AATCCTAAGTGACGACCAGCA
	RV	TCATTGAAGGGGAGATTTGG
sgRNA 1 OFF4	FW	CAGATGTTACCGCTGAGGTG
	RV	CCTTTCCTTCCATATTGTCCA
sgRNA 1 OFF5	FW	TTAAATATTTTTAAGACTTAA
	RV	AACCTATATGAAGAGTCACAT
sgRNA 1 OFF6	FW	TGCAGCTAGCATTTTGGGTA
	RV	CCGAAGTAGCACCAAGAAGG
sgRNA 2 OFF1	FW	GGGAGAAGTGGAGAATGACG
	RV	TTCCTCTCCAGCCACCATAC
sgRNA 2 OFF2	FW	TTGTTCGTCTTGTGTTGCTG
	RV	CATCCCCCACCTTCTCTCT
sgRNA 2 OFF3	FW	GTTCCTACTGGGCGCAACT
	RV	TTCACCGTCTCTCTCCTTCC
sgRNA 2 OFF4	FW	GACCATTAGTGGTGAATTCTTGC
	RV	TCGCTTCTTTCCCTTTTTCTC
sgRNA 2 OFF5	FW	GACATGTTCAAAGGCAAACAAA
	RV	ACCAGATTTGCCTCAACGAC

Table S7. Oligonucleotide primers used for recombinant vector construction, deep sequencing and RT-PCR analysis in these studies.

Primers	Sequence (5'→3')	Purpose
sgRNA1 up	gattGCCTATTTCTCTCAGCCTTA	Vector construction
sgRNA1 down	aaacTAAGGCTGAGAGAAATAGGC	
sgRNA2 up	gattGTCTGCCAAAAGAAAAGAGG	
sgRNA2 down	aaacCCTCTTTTCTTTTGGCAGAC	
pBOsC sgSEQ FW	CAGCTTGGCTCTAGTCGACC	Confirm of sgRNA in pKAtC vector
sgRNA scaffold region	CGGTGCCACTTTTTCAAGTT	
Kanamycin-R Fw	ATGATTGAACAAGATGGATTGCAC	Transgenic plant identification
Kanamycin-R Rv	TCAGAAGAACTCGTCAAGAAGGC	
NGS MS10 1st-Fw	GAATTCCCCAGTACCCCATT	NGS analysis
NGS MS10 1st-Rv	TGCAGCACACACAGTACAAGG	
NGS sg1 2nd-Fw	ACACTCTTTCCCTACACGACGATAATTCAAACAACTCTGAAGAAAGG	
NGS sg1 2nd-Rv	GTGACTGGAGTTCAGACGTGTAATGAAGGTTTTTGATTCATTGC	
NGS sg2 2nd-Fw	ACACTCTTTCCCTACACGACGGACAGTAAAACAACTTTGATCTTTG	
NGS sg2 2nd-Rv	GTGACTGGAGTTCAGACGTGTCATATGTTTCTCCTACCTCCACA	
Ms32 – FW	TGTTTCCATTACCAAGATGC	RT-PCR analysis
Ms32 – RV	GGGGGTTGTGGGGGTAGATT	
SlSTR1 – FW	TGTTTCCATTACCAAGATGC	
SlSTR1 – RV	GGGGGTTGTGGGGGTAGATT	
SlMS10 – FW	AGATCTCTCTGATTCGATTAGCTTCAG	
SlMS10 – RV	TCTTGAAATGGAAGCAACTCAGG	

AtAMS-like – FW	TGCAGAGATGTTATGTTTCAGCATC
AtAMS-like – RV	TCGTCTCTGTCTCTTTCTCCTTCTG
AtMYB103-like – FW	ACAAATTACCTTAGGCCTGATCTCAAACA
AtMYB103-like – RV	AATTCCCATACCAGATAATTTCTTTTTGAG
AtMS1-like-1 – FW	GGGCGTCTTTGCTACAATCCCAAC
AtMS1-like-1 – RV	ATCCATCCTTGATTGCCAACATAATCG
AtTDF1-like1 – FW	GAACGGATAATGATGTGAAGAACCT
AtTDF1-like1 – RV	CTGGTCTAGACATAAATGCACCTTTT
Cysteine protease – FW	ATTGGTGTCGATTGGAGGAAG
Cysteine protease – RV	CAAATGCACTTTCCATAAACCC
Asparatic protease-1 – FW	GTGATATTAATTGGCTTCAATGTGAACC
Asparatic protease-1 – RV	ATACTCGCCGGAACCTGTAACATC
Sister chromatid cohesion – FW	AGTGAGATCATGAGAATTACAGCTCC
Sister chromatid cohesion – RV	GATGAAGTTTGACAGCACTTTCTTG
Endo-1,3-β-glucanase – FW	AAAAAGATTACTACGCGAGTCAAAACATTT
Endo-1,3-β-glucanase – RV	GACGGATCAGGAAGGACAGTAGATTTT
SlACTIN – FW	GGGATGGAGAAGTTTGGTGGTGG
SlACTIN – RV	CTTCGACCAAGGGATGGTGTAGC
